# G protein-coupled receptor kinase 5 deletion suppresses synovial inflammation in a murine model of collagen antibody-induced arthritis

**DOI:** 10.1038/s41598-021-90020-0

**Published:** 2021-05-18

**Authors:** Masakazu Toya, Yukio Akasaki, Takuya Sueishi, Ichiro Kurakazu, Masanari Kuwahara, Taisuke Uchida, Tomoaki Tsutsui, Hidetoshi Tsushima, Hisakata Yamada, Martin K. Lotz, Yasuharu Nakashima

**Affiliations:** 1 Department of Orthopaedic Surgery, Graduate School of Medical Sciences, Kyushu University, 3-1-1 Maidashi, Higashi-ku, Fukuoka City, Fukuoka 812-8582 Japan; 2 Department of Molecular Medicine, The Scripps Research Institute, La Jolla, CA USA

**Keywords:** Inflammation, Rheumatoid arthritis, Experimental models of disease, Monocytes and macrophages

## Abstract

G protein-coupled receptor kinase 5 (GRK5) regulates inflammatory responses via the nuclear factor-kappa B (NF-κB) pathway. This study investigated the functional involvement of GRK5 in the pathogenesis of inflammatory arthritis. Immunohistochemically, rheumatoid arthritis (RA) synovium had a significantly higher proportion of GRK5-positive cells in the synovial lining layer than healthy control synovium. Gene expression and NF-κB activation in lipopolysaccharide-stimulated human SW982 synovial cells were significantly suppressed by silencing of the *GRK5* gene. Similarly, GRK5 kinase activity inhibition in human primary RA synovial cells attenuated gene expressions of inflammatory factors. In a murine model of collagen antibody-induced arthritis, arthritis scores and serum IL6 production of *GRK5* knockout (GRK5^-/-^) mice were significantly lower than those of wild-type mice. Histologically, the degree of synovitis and cartilage degeneration in GRK5^-/-^ mice was significantly lower than in wild-type mice. In in vitro analyses using activated murine macrophages and fibroblast-like synoviocytes, gene expression of inflammatory factors and p65 nuclear translocation were significantly lower in GRK5^-/-^ mice compared to wild-type mice. In conclusion, our results suggested that GRK5 is deeply involved in the pathogenesis of inflammatory arthritis, therefore, GRK5 inhibition could be a potential therapeutic target for types of inflammatory arthritis such as RA.

## Introduction

Macrophages and fibroblast-like cells have been identified to play an important role in the pathogenesis of synovitis in inflammatory arthritis^1–4^. In synovium, macrophages, type A cells, phagocytize actively cell debris, possess an antigen-presenting ability, and produce inflammatory cytokines and chemokines. Fibroblast-like synoviocytes (FLSs), type B cells, also respond to a large variety of chemokines, cytokines, and matrix metalloproteases, and subsequently form a multilayer of synovial membrane. For instance, the morphologic features of FLSs hyperplasia in the synovial lining layer have been described in patients with rheumatoid arthritis (RA)^5,6^. Both macrophages and FLSs significantly contribute to synovial inflammation and joint destruction through nuclear factor-kappa B (NF-κB) signalling^2,7,8^. Since NF-κB signalling regulates various genes involved in inflammation^9–11^, it might be a promising target for treating inflammatory conditions.

Recently, it has been demonstrated that G protein-coupled receptor kinase 5 (GRK5) plays a pathogenic role in the development of osteoarthritis (OA) through catabolic responses in chondrocytes mediated by NF-κB signalling. *GRK5* knockout mice have attenuated cartilage degeneration in a model of surgically-induced OA^12^. GRKs are serine/threonine protein kinases that regulate G protein-coupled receptor (GPCR) signalling^13^. They originally function in the phosphorylation and desensitization of GPCRs^14–16^. Several studies have reported that GRKs can phosphorylate a number of intracellular signalling proteins other than GPCRs^17–19^. Importantly, GRK5 has been reported to promote inflammatory responses by phosphorylating IκBα in the NF-κB signalling pathway^20–22^.

Based on this background, GRK5 was hypothesised to play an important role in the pathogenesis of inflammatory arthritis because of its ability to regulate NF-κB signalling. This study aimed to reveal the characteristics of GRK5 in human synovium and the functional involvement of GRK5 in the development of synovitis in a murine model of collagen antibody-induced arthritis (CAIA).

## Results

### Human RA synovium had significantly more GRK5-positive cells than healthy control tissue

**Figure 1:**
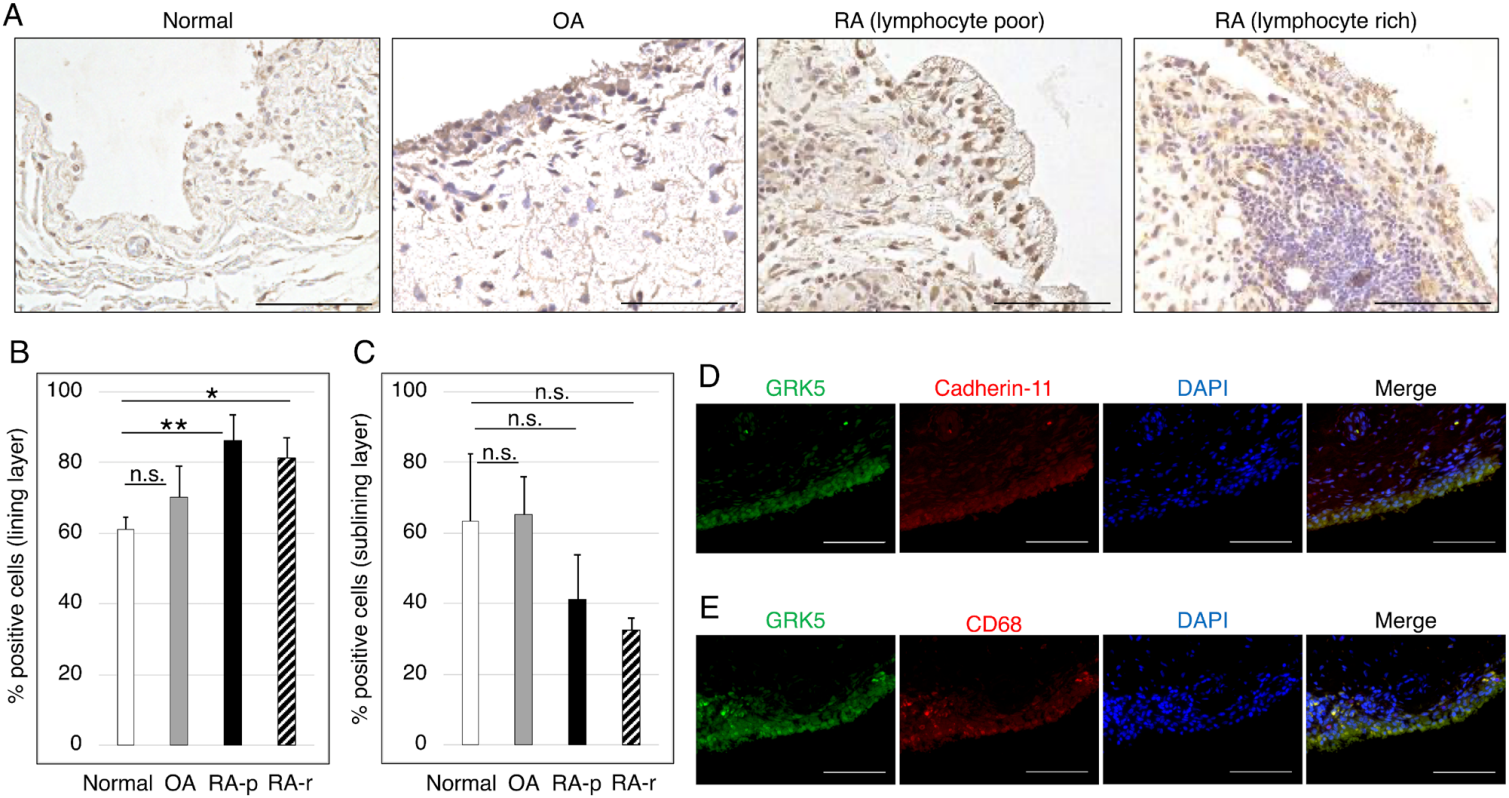
GRK5 expression in human synovium. (**A**) Representative immunohistochemical staining for GRK5 in normal, OA, and RA synovium. RA synovium samples were divided into two groups due to subsynovial inflammatory infiltration; lymphocyte poor (RA-p) and rich (RA-r). Scale bar = 100 μm. (**B**,**C**) Quantification of GRK5-positive cells in the synovial (**B**) lining layer and (**C**) sublining layer of normal, OA, and RA synovium. Results are shown as percentages of cells positive for GRK5 from three normal donors, three OA donors, three RA-p donors, and three RA-r donors. Values are means ± SD. *P < 0.05; **P < 0.01 versus normal. (**D**,**E**) Representative immunofluorescence staining for GRK5, (**D**) Cadherin-11, and (**E**) CD68 in human RA synovium. Scale bar = 100 μm.

The characteristics of GRK5 protein expression in human healthy control (normal), OA, and RA synovium samples were compared. RA synovium were divided into two groups in accordance with subsynovial lymphocyte infiltration; lymphocyte infiltration rich (RA-r) and poor (RA-p). GRK5 protein was strongly expressed in synovial cells, but slightly expressed in lymphocytes of normal, OA, and RA samples (Fig. 1A). In the synovial lining layer, RA synovium had a significant higher frequency of GRK5-positive cells than normal synovium (Fig. 1B). There was no significant difference in GRK5-positive cells between normal, OA, and RA in the synovial sublining layer (Fig. 1C). In RA synovium, both CD68 positive cells, representative of macrophages, and Cadherin-11 positive cells, representative of FLSs, were immune-stained by GRK5 (Fig. 1D,E).

### GRK5 knockdown with siRNA attenuates NF-κB transcriptional activation and the inflammatory gene expression in the human SW982 synovial cell line

**Figure 2:**
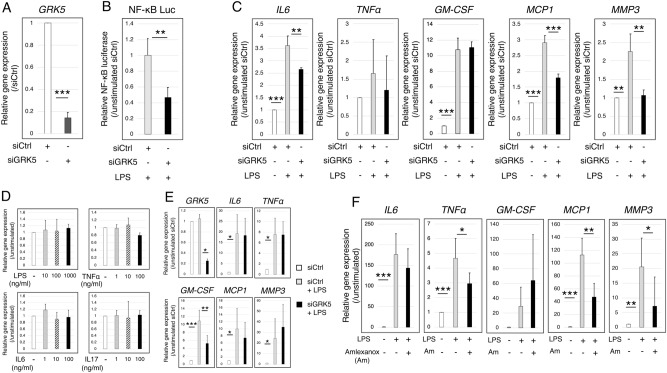
Effects of *GRK5* knockdown by siRNA on NF-κB transcriptional activation and the expression of inflammatory genes in the human synovial SW982 cell line and human primary RA synovial cells. (**A**) Efficiency of *GRK5* knockdown in SW982 cells transfected with small interfering RNA (siRNA)-targeting *GRK5* (siGRK5). Values are means ± SD (n = 3). ***P < 0.001 versus siRNA for control (siCtrl). (**B**) NF-κB transcriptional activation in siGRK5-transfected SW982 cells. Values are means ± SD (n = 4). **P < 0.01 versus siCtrl. (**C**) Changes in gene expressions in response to LPS stimulation in siGRK5-transfected SW982 cells. Values are means ± SD (n = 3). **P < 0.01; ***P < 0.001. (**D**) Effect of various inflammatory stimulation on *GRK5* gene expression in human primary RA synovial cells. Values are means ± SD (n = 3). (**E**) Efficiency of *GRK5* knockdown in human primary RA synovial cells transfected with siGRK5. Values are means ± SD (n = 4). **P < 0.01. (**F**) Efficiency of GRK5 inhibition with Amlexanox. Values are means ± SD (n = 4). *P < 0.05; **P < 0.01.

The functional effects of GRK5 on NF-κB transcriptional activity and gene expressions were analysed in human SW982 cells transfected with small interfering RNA (siRNA) for *GRK5*. SW982, a human synovial cell line, is a useful tool for investigating the expression of inflammatory cytokines or MMPs in response to various stimuli^23^. *GRK5* gene expression was successfully decreased with siRNA (Fig. 2A). Lipopolysaccharide (LPS)-induced NF-κB transcriptional activity was significantly attenuated by *GRK5* knockdown (Fig. 2B). *GRK5* knockdown significantly attenuated LPS-induced gene expressions of *IL6, MCP1,* and *MMP3* compared to siRNA for control (Fig. 2C).

### GRK5 knockdown with siRNA and GRK5 inhibitor attenuates LPS-stimulated the inflammatory gene expression in human primary RA synovial cells

In human primary RA synovial cells, LPS, TNFα, IL6, and IL17 did not affect *GRK5* gene expression (Fig. 2D). *GRK5* knockdown with siRNA significantly attenuated LPS-induced gene expressions of *GM-CSF* compared to siRNA for control (Fig. 2E). Amlexanox, a selective GRK5 inhibitor^24^, was used to analyse the effect of GRK5 kinase activity inhibition. Amlexanox at a concentration of 100 μM significantly suppressed the LPS-induced gene expressions of *TNFα, MCP1*, and *MMP3* (Fig. 2F).

### In a CAIA model, GRK5 knockout mice had less joint swelling, synovitis, and cartilage degradation

**Figure 3:**
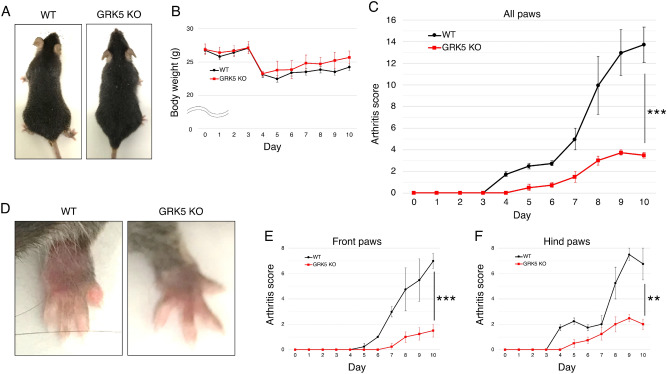
In vivo effects of *GRK5* knockout on joint swelling in a murine model of CAIA. (**A**) Development and growth of WT mice and *GRK5* knockout (KO) mice. (**B**) Changes in body weight over time in *GRK5* KO and WT mice during CAIA. (**C**,**E**,**F**) Changes in arthritis scores over time in *GRK5* KO and WT mice during CAIA. Each leg was assessed using a scale of 0 to 4. (**C**) Total clinical arthritis score ranged from 0 to 16. Arthritis score of (**E**) front paws and (**F**) hind paws ranged from 0 to 8. Values are means ± SD (n = 4). **P < 0.01; ***P < 0.001 versus WT mice. (**D**) Representative macroscopic appearance of *GRK5* KO and WT mice 10 days after CAIA induction.

To investigate the in vivo effect of *GRK5* deletion on inflammatory arthritis, we generated a CAIA model using *GRK5* knockout and wild-type (WT) mice. There were no differences between *GRK5* knockout and WT mice during development and growth (Fig. 3A). After LPS injection on day 3, the body weight of both *GRK5* knockout and WT mice was slightly reduced by the same amount (Fig. 3B). Joint swelling in WT mice started on day 4 and increased over time. On day 10, the arthritis score of WT mice reached 13.8 ± 1.6 (out of 16) (Fig. 3C,D). In contrast, in *GRK5* knockout mice, only minor joint swelling was observed on day 5. The arthritis score on day 10 was 3.5 ± 0.2, which was significantly lower than in WT mice (*p* < 0.001). Divided into front and hind paws, each arthritis score was also significantly lower in *GRK5* knockout mice than in WT mice (Fig. 3E,F).

**Figure 4:**
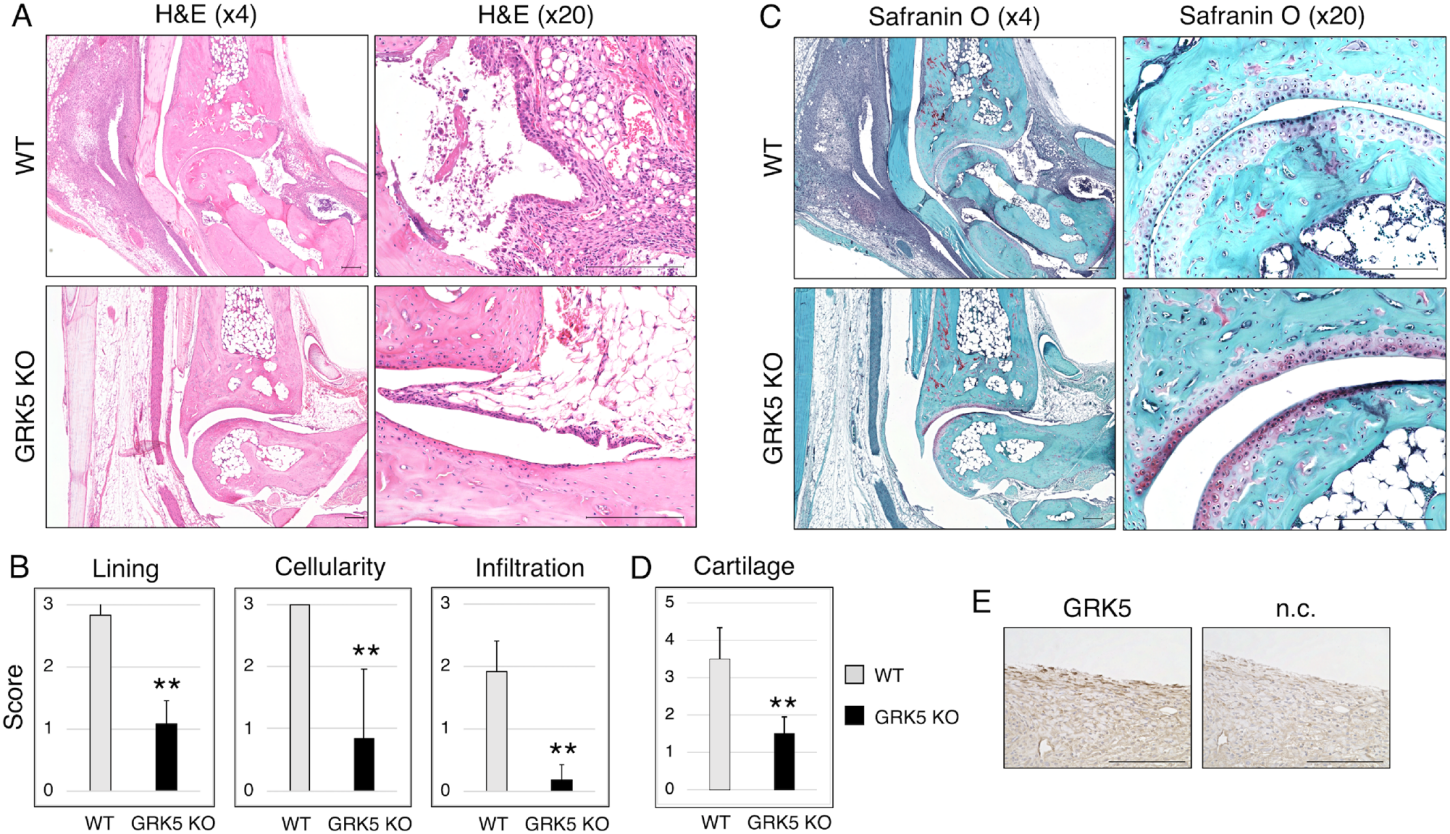
Histological effects of *GRK5* knockout on synovitis and cartilage degradation in a murine model of CAIA. (**A**,**C**) Histological assessment of sagittal murine ankle sections with (**A**) haematoxylin and eosin (**H**,**E**) staining and (**C**) safranin-O and fast green (Safranin O) staining. Scale bars = 200 μm. (**B**) Degree of synovitis in the two groups. The synovitis score divided into three categories using Krenn’s synovitis scoring system. Lining means hyperplasia or enlargement of the synovial lining layer. Cellularity means activation of resident cells or pannus formation. Infiltration means inflammatory infiltration. Values are means ± SD (n = 4). **P < 0.01 versus WT mice. (**D**) The degree of cartilage degradation was compared using Safranin O staining. Values are means ± SD (n = 4). **P < 0.01 versus WT mice. (**E**) Representative immunohistochemical staining for GRK5 in CAIA WT mice. Inflammed synovium in ankle section was stained. Scale bar = 100 μm.

The ankle joint of WT mice on day 10 showed moderate to severe synovitis, such as multilayered lining cell layers, high cellularity, and subsynovial lymphatic infiltration (Fig. 4A). *GRK5* knockout mice had less severe synovitis histologically. *GRK5* knockout mice had significantly lower average synovitis scores than WT mice on day 10 (*p* < 0.01) (Fig. 4B). In the cartilage of WT but not *GRK5* knockout mice, significant loss of Safranin O staining was observed (Fig. 4C). Cartilage degeneration score was significantly lower in *GRK5* knockout mice than in WT mice (*p* < 0.01) (Fig. 4D). In CAIA WT mice, GRK5 was positively immune-stained in the lining cells but not in the sublining cells (Fig. 4E).

### GRK5 knockout suppresses serum IL6 production in CAIA mice and gene expressions of Il6, Tnfα, Il1β, Mip2, and factor B in macrophages

**Figure 5:**
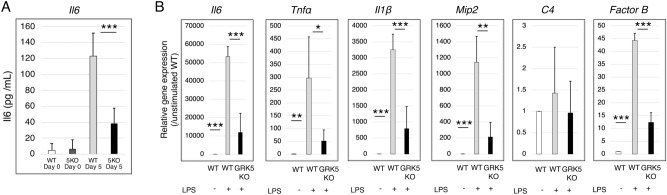
Serum IL6 production after LPS injection in a CAIA model and effects of *GRK5* knockout on the expression of inflammatory genes after LPS stimulation in murine BMDMs. (**A**) Changes in IL6 production after LPS injection in *GRK5* knockout (KO) and WT mice during CAIA. Values are means ± SD (n = 3 per each group). ***P < 0.001 versus WT mice. (**B**) Changes in gene expressions in response to LPS stimulation in *GRK5* KO and WT murine BMDMs. Values are means ± SD (n = 3). *P < 0.05; **P < 0.01; ***P < 0.001.

To explore the effect of *GRK5* knockout on cytokine production in the initiation of CAIA, serum IL6 and TNFα concentration were examined by ELISA. At 2 day after LPS injection, serum IL6 concentration, but not TNFα (data not shown), significantly increased in WT and *GRK5* knockout mice. The increase in serum IL6 concentration significantly suppressed in *GRK5* knockout mice compared to WT mice (*p* < 0.001) (Fig. 5A).

To examine the effect of *GRK5* knockout on gene expressions in macrophages, murine macrophages were derived from bone marrow and differentiated by GM-CSF. Macrophages from *GRK5* knockout mice significantly attenuated LPS-induced gene expressions of *Il6, Tnfα, Il1β, Mip2,* and *factor B* compared to WT mice (Fig. 5B).

### GRK5 knockout attenuates p65 nuclear translocation and gene expressions of Il6, Tnfα, Il1β, Gm-csf, Mcp1, and Mmp3 after LPS stimulation in FLSs

**Figure 6:**
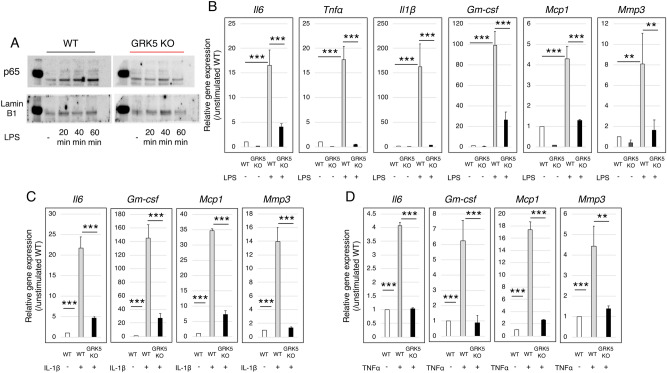
Effects of *GRK5* knockout on p65 nuclear translocation and on the expression of inflammatory genes after inflammatory stimulation in murine FLSs. (**A**) Representative p65 nuclear translocation at 20, 40 and 60 min after LPS stimulation in *GRK5* knockout (KO) and WT murine FLSs. (**B**,**C**,**D**) Changes in gene expressions in response to (**B**) LPS, (**C**) IL-1β, or (**D**) TNFα stimulation in *GRK5* KO and WT murine FLSs. Values are means ± SD (n = 3). **P < 0.01; ***P < 0.001.

p65 nuclear translocation at 20, 40 and 60 min after LPS stimulation increased in FLSs from WT mice. In contrast, FLSs from *GRK5* knockout mice did not show any change in p65 nuclear translocation (Fig. 6A). Consistently, FLSs from *GRK5* knockout mice had significantly attenuated LPS-induced gene expressions of *Il6, Tnfα, Il1β, Gm-csf, Mcp1,* and *Mmp3* compared to WT mice (Fig. 6B). Similarly, gene expression of *Il6, Gm-csf, Mcp1, and Mmp3* were significantly attenuated by knockdown of *GRK5* expression in the presence of IL-1β or TNFα stimulation (Fig. 6C,D).

## Discussion

This study is the first to elucidate the characteristics of GRK5 expression in human synovium and the effect of *GRK5* knockout on the development of arthritis in a murine model of CAIA. GRK5 was more highly expressed in the synovial lining layer of patients with RA than healthy controls. *GRK5* knockdown in a human synovial cell line clearly attenuated LPS-induced expression of inflammatory genes and NF-κB transcriptional activity. Similarly, GRK5 kinase activity inhibition in human primary RA synovial cells suppressed LPS-induced inflammatory gene expressions. In a CAIA model, *GRK5* knockout mice had significantly suppressed development of arthritis, with less severe synovitis and cartilage degeneration compared to WT mice. Suppressed serum IL6 production seemed as a causal phenomenon for less severity of CAIA in *GRK5* knockout mice. Consistently, murine macrophages from *GRK5* knockout mice had downregulated gene expression of *Il6, Tnfα, Il1β, Mip2, and factor B*. In FLSs from *GRK5* knockout mice, downregulated inflammatory gene expression was accompanied by attenuation of p65 nuclear translocation. These results suggested that GRK5 plays a pathogenic role in the development of synovitis in inflammatory arthritis via NF-κB signalling.

In general, GRKs consist of seven isoforms that share structural and functional similarities in the phosphorylation and desensitisation of GPCRs^25^. GRK2, GRK3, GRK5, and GRK6 are ubiquitously found in cells throughout the body. In contrast, GRK1, GRK4, and GRK7 have limited tissue distribution^26,27^. Lack of *GRK2* expression results in embryonic lethality due to cardiac abnormalities^28^, but mice with knockouts of other GRKs develop normally.

Importantly, GRKs have been reported to have disease-specific functions by interacting with intracellular substrates other than GPCRs^29–31^. In particular, GRK5 has been reported to activate NF-κB signalling through IκBα phosphorylation and p65 nuclear translocation^12,20,32^. In macrophages, Patial et al*.* demonstrated that GRK5 is a positive regulator of the TLR4-induced IκBα-NFκB pathway as well as a key modulator of LPS-induced inflammatory responses^20^. In myocytes, overexpression of GRK5 increases the levels of NF-κB p50 and p65 in vitro and in vivo, whereas loss of GRK5 expression resulted in lower cardiac NF-κB levels^32^. In chondrocytes, GRK5 overexpression significantly increases NF-κB transcriptional activation in humans and GRK5 deletion reduces IκBα phosphorylation and p65 nuclear translocation in mice^12^. Consistent with these findings, the present study showed that active synovial lining cells from patients with RA expressed substantial amounts of GRK5 protein, and LPS-induced NF-κB transcriptional activity and p65 nuclear translocation in synovial cells were significantly attenuated by downregulation of *GRK5*.

In this study, the murine CAIA model was used as a model of arthritis because it has provided the most reproducible induction of synovitis and consequent cartilage degradation in the study of inflammatory arthritis^33–35^. The collagen-induced arthritis (CIA) model is another traditional murine model of arthritis, but there is a lower incidence of arthritis in C57BL/6 background mice. The in vivo results from this study demonstrated that severe arthritis can be successfully induced in a CAIA model, as determined by the macroscopic score and histological scores of synovium and cartilage in WT mice. Previously, Tarrant et al*.* examined the effect of *GRKs* knockout on the pathogenesis of arthritis in the K/BxN model in which a spontaneously erosive arthritis with similarities to RA occurs^36^. In their study, *GRK6* and *GRK2* knockout mice demonstrated severe arthritis and weight loss due to the loss of anti-chemotaxis property during the development of the K/BxN model, whereas there was no significant difference in the response to K/BxN serum-transfer between *GRK5* knockout and WT mice. Accordingly, *GRK5* knockout did not show any significant phenotype in the K/BxN arthritis model. Although there is no clear explanation for the discrepancy with our findings, the difference in the pathogenesis between the CAIA and the K/BxN model might be a potential influential factor. CAIA, but not K/BxN model, needs a LPS-injection to induce arthritis after sensitization by antibody. In our study, LPS significantly increased NF-κB transcriptional activation, therefore, *GRK5* deletion contributed to suppress inflammatory responses via NF-κB signaling.

Primary activation of innate immune cells including macrophages plays an pivotal role in the development of CAIA based on functional complement system^37^. In the present study, *GRK5* knockout markedly attenuated serum IL6 concentration after LPS injection at the initiation of CAIA. The in vitro results from this study confirmed that macrophages from *GRK5* knockout mice showed a less LPS-response in gene expressions of inflammatory factors. In complement elements, not *C4* but *factor B* gene expression was significantly attenuated in *GRK5* knockout macrophages, indicating that alternative complement pathway, which is essential in the pathogenesis of CAIA^37^, was involved. Collectively, these findings suggested that GRK5 functions as a positive intermediator of LPS-stimulation in macrophages, therefore, *GRK5* knockout significantly delays the onset of arthritis and suppresses the initial progression down to a mild level in a CAIA.

FLSs, also termed type B synoviocytes, are the most abundant resident cell type in synovial tissue^38^. Numerous lines of evidence support the potential contribution of FLSs to the pathogenesis of inflammatory arthritis^3,4,39,40^. Activated FLSs are responsible for excessive matrix degradation, which destroys cartilage and causes permanent joint damage in RA^41–43^. The in vitro results of this study showed that not only LPS-induced but also IL-1β and TNFα-induced inflammatory cytokines and chemokines are markedly attenuated in FLSs from *GRK5* knockout mice, a possible mechanism by which *GRK5* knockout results in less severe joint destruction in a murine CAIA model.

Regarding other disorders, in the pathogenesis of OA, *GRK5* knockout mice have less cartilage degradation compared to WT mice in a model of surgically induced OA. Chondrocytes in *GRK5* knockout mice have a smaller response to IL-1β stimulation^12^. In the pathogenesis of sepsis, Packiriswamy et al*.* reported that mortality due to induced polymicrobial sepsis was prevented in *GRK5* knockout mice; therefore, GRK5 is an important regulator of inflammation in polymicrobial sepsis^44^. Research in the field of tumour biology showed that GRK5 phosphorylates p53 and inhibits DNA damage-induced apoptosis in cultured osteosarcoma cells and mice^45^. In the heart, *GRK5* overexpression was reported to worsen heart failure and cardiac hypertrophy by functioning as a nuclear HDAC kinase irrespective of GPCRs^46,47^.

This study has some limitations. First, only inflammatory arthritis was included in the CAIA model, although there are several models of arthritis such as CIA, adjuvant-induced arthritis, and glucose-6-phosphate isomerase-induced arthritis. Second, the effect of *GRK5* knockout in this murine CAIA model was evaluated at two time points, 5 and 10 days after CAIA induction. Additional analysis at different time points might provide further information about the functional effects of GRK5 on the pathogenesis of arthritis. Third, *GRK5* knockout mice used in this study were global knockout mice. FLSs-specific and macrophages-specific *GRK5* knockout animals would be useful to analyze further specific function of GRK5.

In conclusion, our results demonstrated that GRK5 protein is highly expressed in inflammatory synovium and GRK5 deletion suppresses inflammatory responses in vivo and in vitro. GRK5 is a positive regulator of inflammatory responses; therefore, GRK5 inhibition could be a potential therapeutic target for types of inflammatory arthritis such as RA.

## Materials and methods

### Ethical approval

All animal studies were approved by the Committee of Ethics on Animal Experimentation of the Faculty of Medicine, Kyushu University (Fukuoka, Japan) and carried out in accordance with relevant rules and regulations. All animal studies were also carried out in compliance with the ARRIVE guidelines. Human synovium from individuals were obtained from healthy controls at autopsy under approval of the Scripps Human Subjects Committee or patients undergoing total knee arthroplasty under the approval of the Ethics Committee of Kyushu University Hospital. Informed consent was obtained from all patients prior to surgery. All experimental procedures were performed under the guidelines of Kyushu University (Fukuoka, Japan).

### Clinical samples

Human normal synovium was harvested from three donors (aged 21–47 years, mean ± SD = 37.7 ± 14.5 years) with no history of joint disease. The inflammatory grade was 0. Human OA synovium samples were obtained from three donors (aged 60–76 years, mean ± SD = 67.7 ± 8.0 years) with grade III–IV OA. Human RA synovium samples were obtained from six donors (aged 37–66 years, mean ± SD = 56.5 ± 12.9 years). All patients with RA satisfied the 2010 American College of Rheumatology criteria for RA^48^.

### Immunohistochemistry of human synovium samples

Immunohistochemistry was performed on all human synovium tissue sections. Synovium samples were fixed in 4% paraformaldehyde for 2 days, delipidised, cut into sections that were 4 μm thick, and embedded in paraffin. Antigen retrieval was performed overnight with 1 mM EDTA at pH 8.0. Endogenous peroxidase activity was blocked with 3% hydrogen peroxidase in methanol for 30 min. For the blocking procedure, each specimen was placed in normal horse serum (VECTASTAIN Universal Elite Kit; Vector Laboratories, Burlingame, CA) for 20 min and then incubated for 1 h at room temperature with primary anti-GRK5 antibody (Proteintech, Rosemont, IL). Finally, the samples were counterstained with haematoxylin.

### Quantification of positive cells in human synovium samples

GRK5 localisation in each synovial layer was systematically assessed by counting the number of positive cells. The frequency of positive cells was expressed as a percentage relative to the total number of cells counted in each layer with the BZ-II Analyzer software program (Keyence).

### Immunofluorescence staining of human RA synovium samples

Sections were stained with primary antibodies at room temperature for 1 h, then incubated with Alexa Fluor-conjugated secondary antibodies (Thermo Fisher Scientific, Waltham, MA). The following primary antibodies were used: GRK5 (Abcam and Proteintech), Cadherin-11 (GENETEX), and CD68 (Dako).

### Human RA synovial cells isolation and culture

Tissue samples were minced into pieces and incubated for 2 h at 37 °C in 5% CO_2_ with 4 mg/mL of collagenase (Collagenase; Wako, Osaka, Japan) in DMEM/F12 (Gibco, Langley, OK). Dissociated cells were centrifuged at 500 g and re-suspended in DMEM containing 10% FBS (Gibco). The medium was changed every 3–4 days, and remaining adherent cells were used for subsequent experiments.

### siRNA transfection

We used human SW982 cells and human primary RA synovial cells for following procedures. Cells were seeded in 12-well plates at a density of 0.5 × 10^5^ cells/well with DMEM and 10% FBS. After the cells reached subconfluence, they were transfected with siRNA (5 nM) against GRK5 (siGRK5, Santa Cruz Biotechnology, Dallas, TX, USA) for 6 h using Lipofectamine RNAiMAX (Thermo Fisher Scientific). Thirty-six hours after transfection, cells were serum-starved for 12 h and then stimulated with LPS (1 μg/mL) for 6 h.

### Total RNA extraction and quantitative real-time RT-PCR

Total RNA was extracted from human SW982 cells, human primary RA synovial cells, murine macrophages, and murine FLSs using the TRIzol reagent (Thermo Fisher Scientific). Total RNA was reverse-transcribed to cDNA using the PrimeScript RT reagent kit (Takara Bio, Kusatsu, Japan). Quantitative real-time RT-PCR was performed using the Light Cycler 2.0 System (Roche, Basel, Switzerland) and SYBR Premix EX Taq II (Takara Bio). Respective dates were normalised against the corresponding levels of mouse *18 s rRNA* or human *GAPDH*, a housekeeping gene. The primers are summarized in Supplementary Table S1 online.

### Luciferase assay

Human SW982 cells were simultaneously transfected with siRNA, the pNL3.2 (NlucP/NF-κB-RE/Hygro; Promega, Madison, WI) vector, and the pGL-CMV (luc2/CMV/Neo; Promega) vector using Lipofectamine 3000 (Thermo Fisher Scientific). Thirty-six hours after transfection, cells were serum-starved for 12 h and then stimulated with LPS (1 μg/mL) for 6 h. Lysates were prepared and analysed using the Dual-Luciferase System (Promega).

### Amlexanox treatment of human RA synovial cells

Human primary RA synovial cells were treated for 3 h with 100 μM amlexanox (MedChem Express), previously identified to be a GRK5 inhibitor^24^. After amlexanox treatment for 3 h, cells were stimulated with LPS (1 μg/mL) for 6 h.

### Mice

*GRK5* knockout mice and WT mice with a C57BL/6 background were used in all animal experiments. These *GRK5* knockout mice were general knockout mice. Mice were housed in a specific pathogen-free facility with a 12-h light, 12-h dark cycle and given free access to food and water. We have previously reported that there were no differences during growth and development between *GRK5* knockout and WT mice^12^.

### CAIA in mice

Both *GRK5* knockout and WT mice were used with the same protocol. As male mice have been reported to have greater susceptibility to CAIA induction than female mice^49^, we only used male mice in this study. Male mice were injected intraperitoneally with a five-clone cocktail of collagen type II antibodies (5 mg/mouse; Chondrex, Redmond, WA, USA) to induce arthritis at 12 weeks of age. Three days after antibody administration, 25 μg of LPS were injected intraperitoneally. Daily clinical scoring was undertaken using the manufacturer’s protocol. The score for each leg was assessed on a scale of 0 to 4 (0, no change; 1, swelling and redness of one joint; 2, moderate swelling and erythema of ≧ two joints; 3, severe swelling and erythema of all joints; 4, extensive swelling and deformity of all joints); the total clinical arthritis score ranged from 0 to 16^34,50^. Arthritis was initially observed on day 4 and peaked around day 9–10. Thus, all mice were sacrificed on day 10, and paws were collected (n = 4 per each group).

Paws were fixed in 4% paraformaldehyde for 2 days. After decalcification, murine ankle joints were cut into sections that were 4 μm thick along the sagittal plane and embedded in paraffin. Next, sagital ankle sections were stained with haematoxylin and eosin (H&E) and safranin-O and fast green (Safranin O) (n = 4 per each group). The severity of synovitis was quantified using a histopathological synovitis grading system previously reported by Krenn *et al*^51^. Cartilage degeneration (decrease in Safranin O) was quantified using the following semiquantitative grading scale: 0 = no pathological changes; 1 = minimal (minimal changes, or lesions involving < 25% of the whole section); 2 = slight (obvious changes, or lesions affecting 25–50% of the whole section; 3 = moderate (relatively severe changes, or lesions involving 50–75% of the whole section); 4 = severe (very severe changes, or lesions affecting > 75% of the whole section)^52^. Both scores were evaluated by two blinded independent observers. Scores were averaged to minimise observer bias. Immunohistochemistry was also performed on CAIA ankle sections.

### Serum preparation and enzyme-linked immunosorbent assay (ELISA)

In a CAIA model, murine blood samples were collected at day 0 (before a five-clone cocktail of collagen type II antibodies injection) and day 5 (paws swelling started to be appeared after LPS injection) (n = 3 per each group). Serum was prepared by centrifugation of coagulated blood samples at 3,000 rpm for 15 min and frozen at − 80 °C. The concentrations of IL6 and TNFα in collected serum samples were measured using murine IL6 and TNFα ELISA kits (R&D Systems, Minneapolis, MN, USA).

### Murine bone marrow derived macrophages (BMDMs) culture

Murine BMDMs were obtained from both *GRK5* knockout and WT mice. Whole bone marrow was flushed from the femurs and tibiae, and cells were collected by centrifugation at 400 g for 5 min at 4 °C. Cells were resuspended in RPMI 1640 (Gibco) supplemented with 10% FBS and 20 ng/ml murine recombinant GM-CSF (R&D Systems), then seeded and cultured at 37 °C in 5% CO2 for 7 days. Non-adherent cells were removed by washing, and only adherent cells were used for subsequent experiments.

### Murine FLSs isolation and culture

Tissue samples were minced into pieces (1–3 mm) and incubated for 2 h at 37 °C in 5% CO_2_ with 4 mg/mL of collagenase in DMEM/F12. Dissociated cells were centrifuged at 500 g and re-suspended in DMEM containing 10% FBS. After 24 h, non-adherent cells were removed. The remaining adherent cells were cultured in DMEM with 10% FBS. Cultures were maintained at 37 °C and 5% CO_2_. The medium was changed every 3–4 days. FLSs from passages 3 to 8 were used for subsequent experiments. Murine FLSs were isolated from hip joint tissue from both *GRK5* knockout and WT mice as previously described^53^.

### Western blotting

Experiments were carried out as previously described^54^. Nuclear and cytoplasmic extracts were isolated using nuclear and cytoplasmic extraction reagents (Thermo Fisher Scientific). Cell lysates were electrophoresed in 4–12% gradient polyacrylamide gels (Invitrogen), and the resolved proteins were transferred to nitrocellulose membranes (Amersham Biosciences, Arlington Heights, IL, USA). Membranes were blocked with blocking buffer (Takara Bio), washed in Tris-buffered saline with Tween (TBST), and incubated with primary antibodies against p65 (Cell Signaling Technology) and Lamin B1 (Abcam) diluted 1:1000 in Can Get Signal Immunoreaction Enhancer Solution 1 (TOYOBO, Osaka, Japan). Prior to hybridisation with primary antibodies, membranes were cut at the each expected blots point. After washing in TBST, secondary anti–rabbit IgG antibodies (Cell Signaling Technology) were added. Immunoreactivity was detected with ECL Prime (Amersham Biosciences) and photographed on an Ez Capture MG (ATTO, Tokyo, Japan). Band densities were calculated using CS Analyzer 3.0 (ATTO).

### Statistical analysis

All experiments were repeated at least three times. All values are represented as means ± SD. Student’s *t*-test was used for two-groups comparisons. Tukey–Kramer test was used for multiple comparisons. All data analyses were performed using JMP 13 statistical software (SAS Institute, Cary, NC, USA). *P*-values less than 0.05 were considered significant.

### Patient consent

Obtained.

### Provenance and peer review

Not commissioned; externally peer reviewed.

## Supplementary information

Supplementary Information.

## Data Availability

The datasets generated and analysed during the current study are available from the corresponding author on reasonable request. All data generated or analysed during this study are included in this published article (and its Supplementary Information files).
