# Supplementary material for: G protein-coupled receptor kinase 5 deletion suppresses synovial inflammation in a murine model of collagen antibody-induced arthritis
Source: Sci Rep. 2021 May 18;11:10481. doi: 10.1038/s41598-021-90020-0 (PMC8131379; doi:10.1038/s41598-021-90020-0)
Supplement: Supplementary file 1 — Supplementary Information. [file 41598_2021_90020_MOESM1_ESM.pdf]

Supplementary information

G protein-coupled receptor kinase 5 deletion suppresses synovial inflammation in a murine model of collagen antibody-induced arthritis.

Masakazu Toya[1], Yukio Akasaki[1], Takuya Sueishi[1], Ichiro Kurakazu[1], Masanari Kuwahara[1], Taisuke Uchida[1], Tomoaki Tsutsui[1], Hidetoshi Tsushima[1], Hisakata Yamada[1], Martin K. Lotz[2], Yasuharu Nakashima[1]

1 Department of Orthopaedic Surgery, Graduate School of Medical Sciences, Kyushu University, Fukuoka, Japan

2 Department of Molecular Medicine, The Scripps Research Institute, CA, USA

Corresponding author: Yukio Akasaki

Department of Orthopaedic Surgery, Graduate School of Medical Sciences, Kyushu University, 3-1-1 Maidashi, Higashi-ku, Fukuoka city, Fukuoka, 812-8582, Japan.

E-mail: akasaki@ortho.med.kyushu-u.ac.jp

Tel: +81 926425488

Supplemental Table S1. Primers for real-time PCR.

Genes	Strand	Primer sequences	Origin
GRK5	S<br>AS	5′- TCAACAGCCAGTTTGTGGTCAA -3′<br>5′- GATCTTCACGGCCAGTCCGA -3′	Human
IL6	S<br>AS	5′- GGTACATCCTCGACGGCATCT -3′<br>5′- GTGCCTCTTTGCTGCTTTCAC -3′	Human
TNFα	S<br>AS	5′- GCCGCATCGCCGTCTCCTAC -3′<br>5′- AGCGCTGAGTCGGTCACCCT -3′	Human
GM-CSF	S<br>AS	5′- AAATGTTTGACCTCCAGGAGCCGA -3′<br>5′- AGGTGATAATCTGGGTTGCACAGG -3′	Human
MCP1	S<br>AS	5′- CATTGTGGCCAAGGAGATCTG -3′<br>5′- CTTCGGAGTTTGGGTTTGCTT -3′	Human
MMP3	S<br>AS	5′- CTGGCCTGCTGGCTCATGCTT -3′<br>5′- GCAGGGTCCTTGGAGTGGTCA -3′	Human
GAPDH	S<br>AS	5′- GGTGAAGGTCGGAGTCAACGGA -3′<br>5′- GAGGGATCTCGCTCCTGGAAGA -3′	Human
Grk5	S<br>AS	5′- AAACACTTTCCGGCAGTACC -3′<br>5′- GGCCATAATCATCCAGCAAG -3′	Mouse
Tnfα	S<br>AS	5′- GACCCTCACACTCAGATCATCTTCT -3′<br>5′- CCTCCACTTGGTGGTTTGCT -3′	Mouse
Il1β	S<br>AS	5′- ATGCCACCTTTTGACAGTGATG -3′<br>5′- AGCTTCTCCACAGCCACAAT -3′	Mouse
Il6	S<br>AS	5′- CCGGAGAGGAGACTTCACAG -3′<br>5′- TCCAGTTTGGTAGCATCCATC -3′	Mouse
Gm-csf	S<br>AS	5′- CCAGCTCTGAATCCAGCTTCTC -3′<br>5′- TCTCTCGTTTGTCTTCCGCTGT -3′	Mouse
Mcp1	S<br>AS	5′- CTTCTGGGCCTGCTGTTCA -3′<br>5′- CCAGCCTACTCATTGGGATCA -3′	Mouse
Mmp3	S<br>AS	5′- GCTGCCATTTCTAATAAAGA -3′<br>5′- GCACTTCCTTTCACAAAG -3′	Mouse
Mip2	S<br>AS	5’- ATCCAGAGCTTGAGTGTGACGC -3’<br>5’- AAGGCAAACTTTTTGACCGCC -3’	Mouse

C4	S<br>AS	5’- TGCTGAAGATTCTGAGTTTGGC -3’<br>5’- CAGCTAGCCGTCTCCTGCA -3’	Mouse
Factor B	S<br>AS	5’- GGCAACAGCTGGTACCCTCTT -3’<br>5’- CCTTTAGCCAGGGCAGCAC -3’	Mouse
18SrRNA	S<br>AS	5´- GTAACCCGTTGAACCCCATT -3´<br>5´- CCATCCAATCGGTAGTAGCG -3´	Mouse

S, sense; AS, antisense.
